# Supplementary material for: Subspecies in the global human gut microbiome
Source: Mol Syst Biol. 2017 Dec 14;13(12):960. doi: 10.15252/msb.20177589 (PMC5740502; doi:10.15252/msb.20177589)
Supplement: Supplementary file 1 — Appendix [file MSB-13-960-s001.pdf]

# Appendix

## Contents

Appendix Figure S1: Horizontal coverage of compared genomes ........ 2

Appendix Figure S2: Subspecies exclusivity ........ 3

Appendix Figure S3: Domination of one subspecies per species within individuals ........ 4

Appendix Figure S4: Accuracy of SC and SSSC reconstruction ........ 6

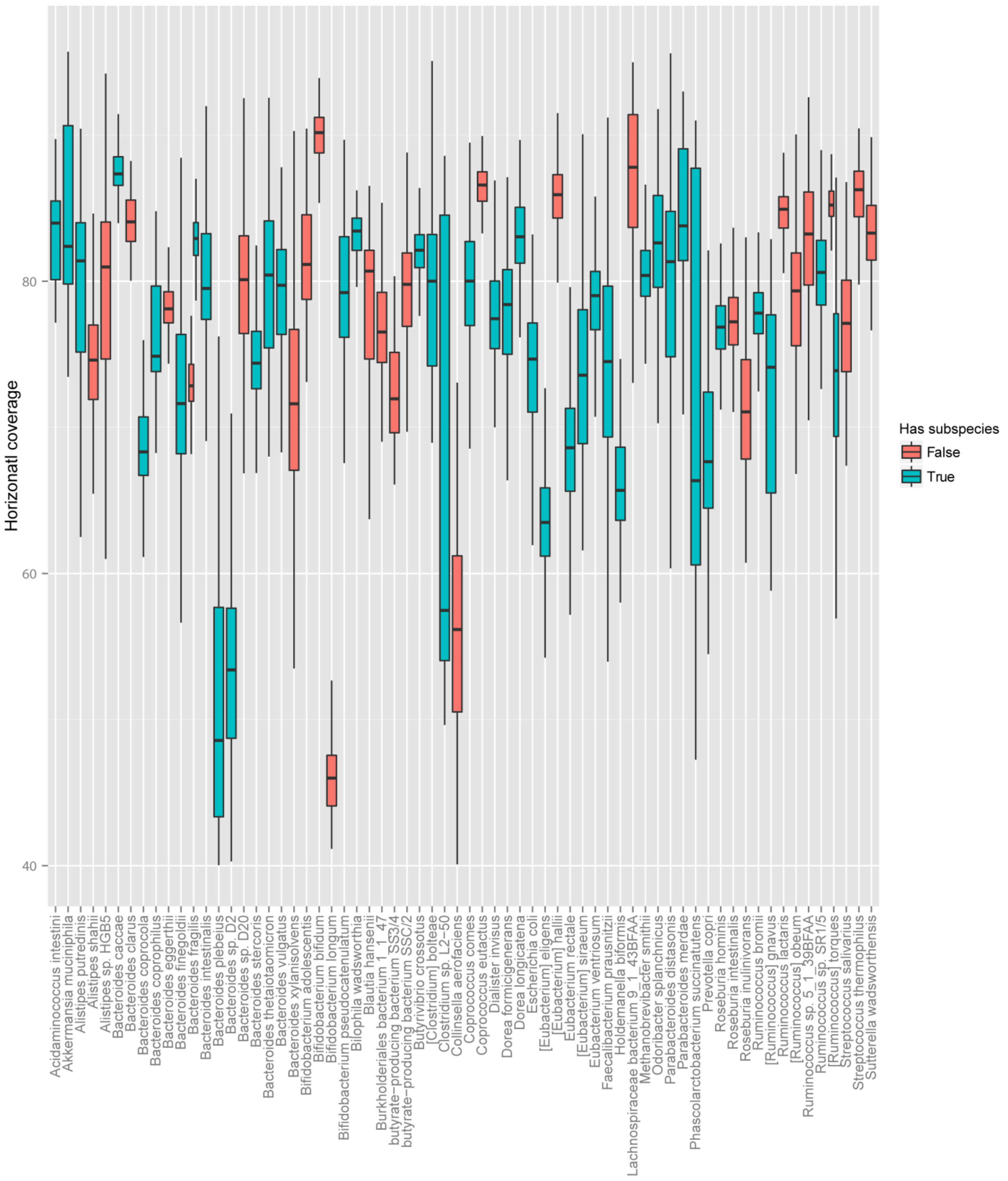


## Appendix Figure S1: Horizontal coverage of compared genomes

The distribution of horizontal coverage per sample, over the considered genomes, shows that for the vast majority of species more than 70% of the genome is covered. Thus, pair-wise comparisons between samples are a good estimate of overall distance.

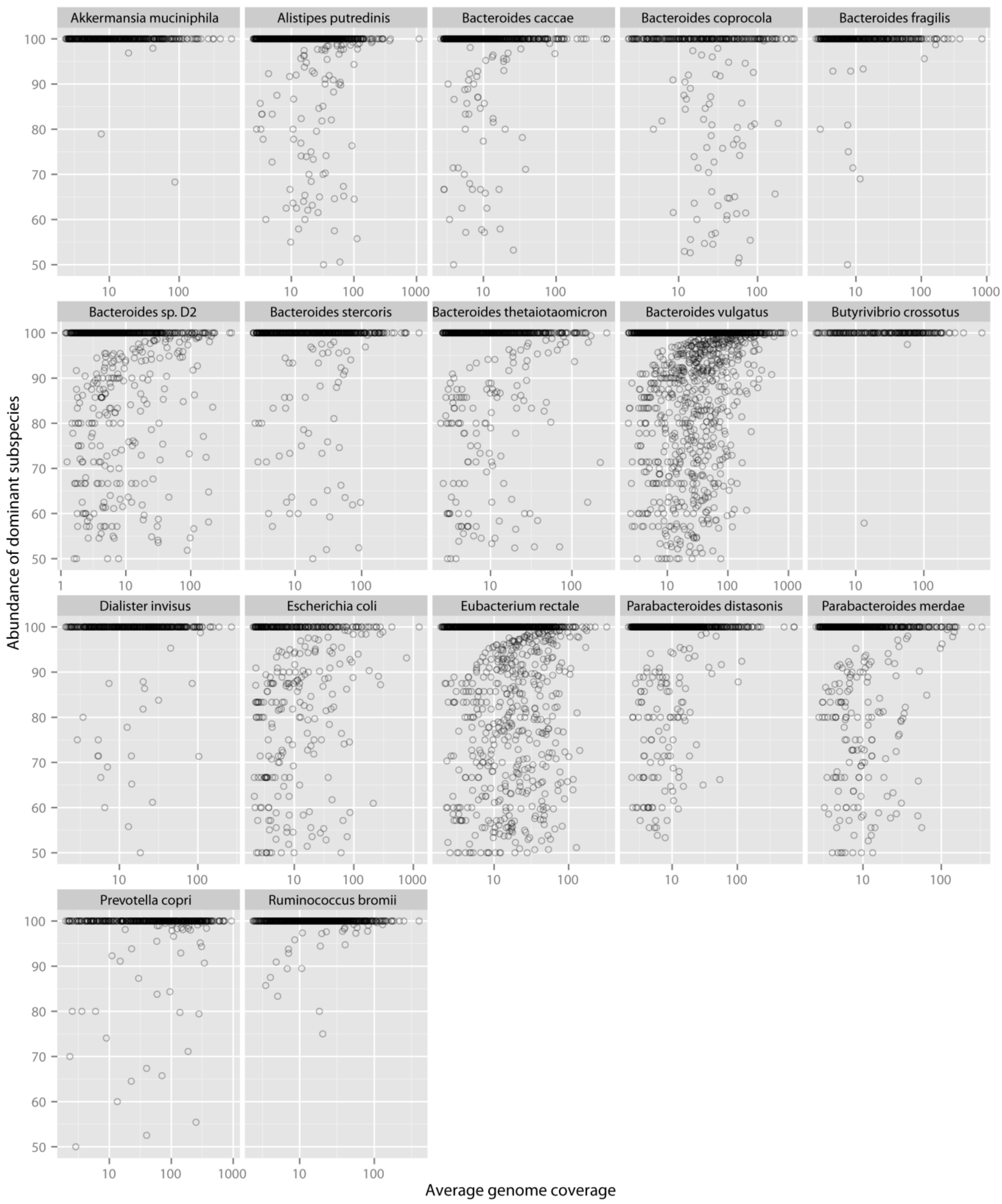


## Appendix Figure S2: Subspecies exclusivity

For species in which at least 50 samples exist with a coverage more than 50x, we illustrate the frequency of the dominant subspecies within these samples (based on the median frequency of marker alleles determined for these subspecies). There is no observable effect of coverage on observed exclusivity. That is, even at more than 1000x, we observe the dominant subspecies in a sample to have a median allele frequency of 100%.

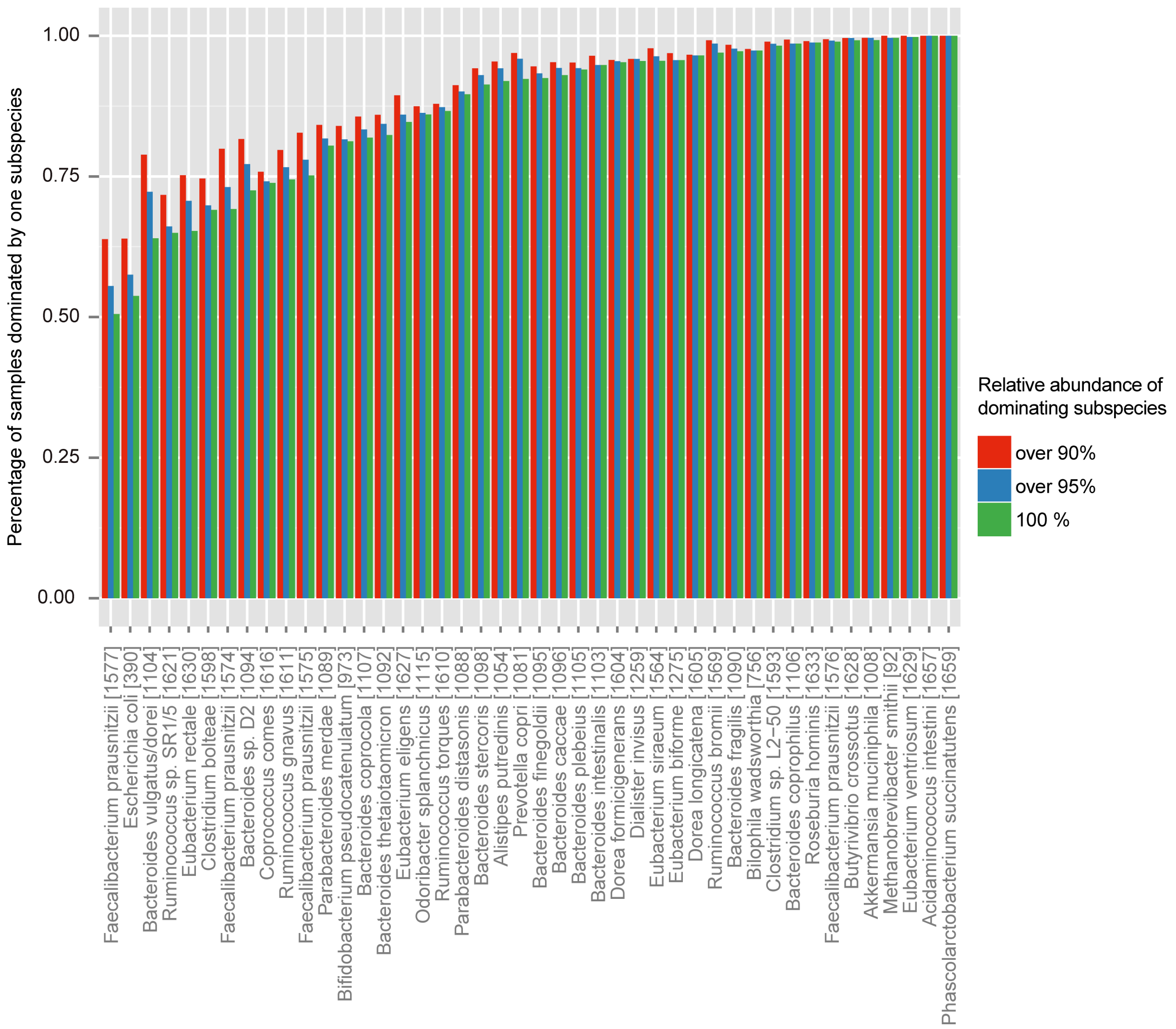


## Appendix Figure S3: Domination of one subspecies per species within individuals

For most species, more than 80% of individuals are dominated by only one con-specific subspecies even when applying several minimum frequencies to define “dominating subspecies”, ranging from a frequency of 90% to 100%.

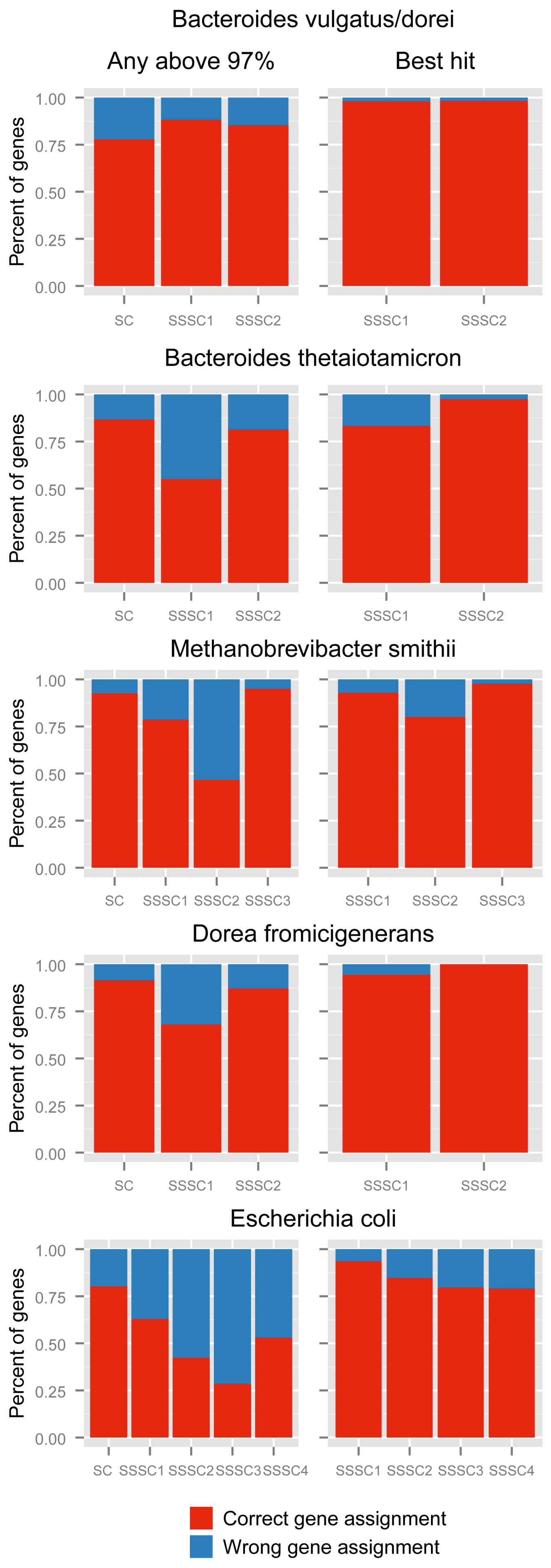

Bacteroides vulgatus/dorei
Any above 97%
Best hit
Percent of genes
1.00
0.75
0.50
0.25
0.00
SC
SSSC1
SSSC2
Bacteroides thetaiotamicron
Methanobrevibacter smithii
SSSC3
Dorea fromicigenerans
Escherichia coli
SSSC4
Correct gene assignment
Wrong gene assignment

## Appendix Figure S4: Accuracy of SC and SSSC reconstruction

We benchmarked the accuracy of our correlation-based reconstruction of subspecies-specific gene pools for five species for which multiple representative genomes were available as a ground truth. To determine whether SSSC genes were assigned to the correct subspecies, we used a BLAST best-hit approach, where a gene (from the metagenomics catalogue) is assigned to the subspecies genome to which it has highest similarity; according to this assessment, our correlation-based SSSC reconstruction is highly accurate in assigning genes to the correct subspecies. To benchmark SC versus SSSC assignments, we BLASTed metagenomics genes within a 97% identify cutoff against the respective reference genomes. If a gene matches multiple subspecies genomes at this cutoff we consider it to belong to SC otherwise to belong to the SSSC of the subspecies genome it exclusively matches. This evaluation shows a good recovery of the respective gene pools, but also reflects varying genomic dissimilarity between subspecies for which this 97% identity cutoff as well as the 95% similarity clustering of the metagenomics gene catalogue (*52*) may not be optimal.
