# Supplementary material for: Subspecies in the global human gut microbiome
Source: Mol Syst Biol. 2017 Dec 14;13(12):960. doi: 10.15252/msb.20177589 (PMC5740502; doi:10.15252/msb.20177589)
Supplement: Supplementary file 2 — Expanded View Figures PDF [file MSB-13-960-s002.pdf]

Expanded View Figures

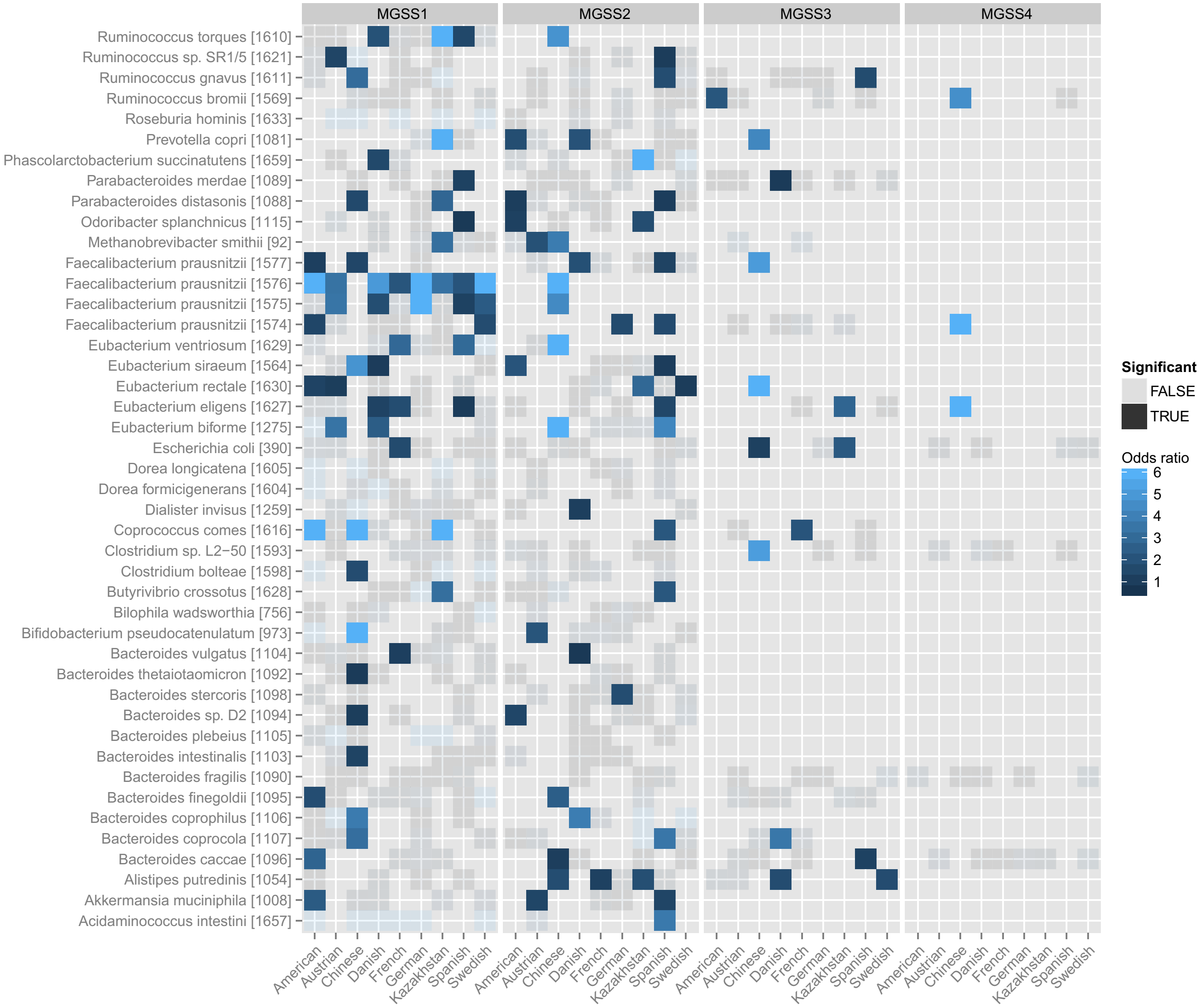

Figure EV1. Geographic distribution of subspecies.

The enrichment of each MGSS is computed for each species within each country, showing which subspecies is enriched where. An enrichment is considered significant if the *P*-value of Fisher's exact test is less than 0.05.

© 2017 European Molecular Biology Laboratory

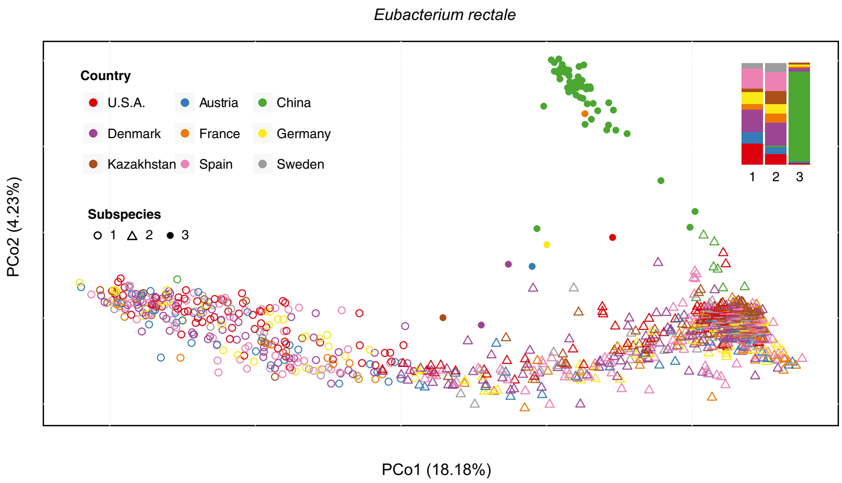

Figure EV2. Geographic patterns of *Eubacterium rectale* subspecies.

The PCoA of nucleotide variation shows a clear geographic pattern of *Eubacterium rectale*, with *MGSS3* being almost exclusively found in the Chinese population. The relative proportion of countries in each subspecies is summarized in the top right bar plot highlighting that very few individuals from the other countries considered in the study harbor *MGSS3*.

© 2017 European Molecular Biology Laboratory

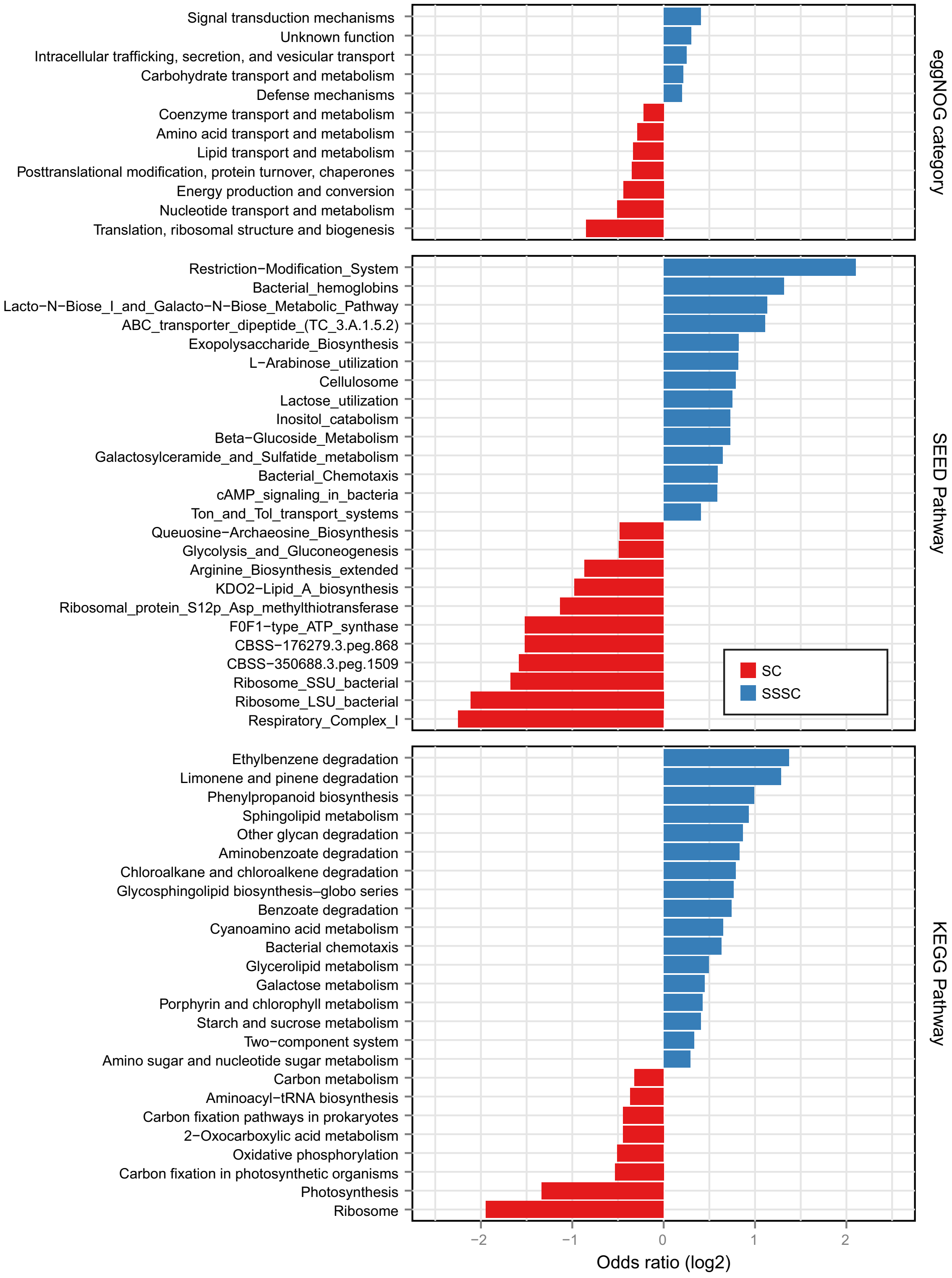

Figure EV3. Functional category enrichments between SC and SSSC.

Analyzing the species core (SC) and subspecies-specific core (SSSC) for enrichment of gene functional categories, as derived from three different functional annotation databases, we find an SC enrichment of basic functions related to translation, ribosomal process, central energy production, and glucose metabolism, while auxiliary functions, including metabolism of complex carbohydrates, lipids, and aromatic compounds, which are potentially under selection by the environment, tend to be enriched in the subspecies-specific core.

 © 2017 European Molecular Biology Laboratory

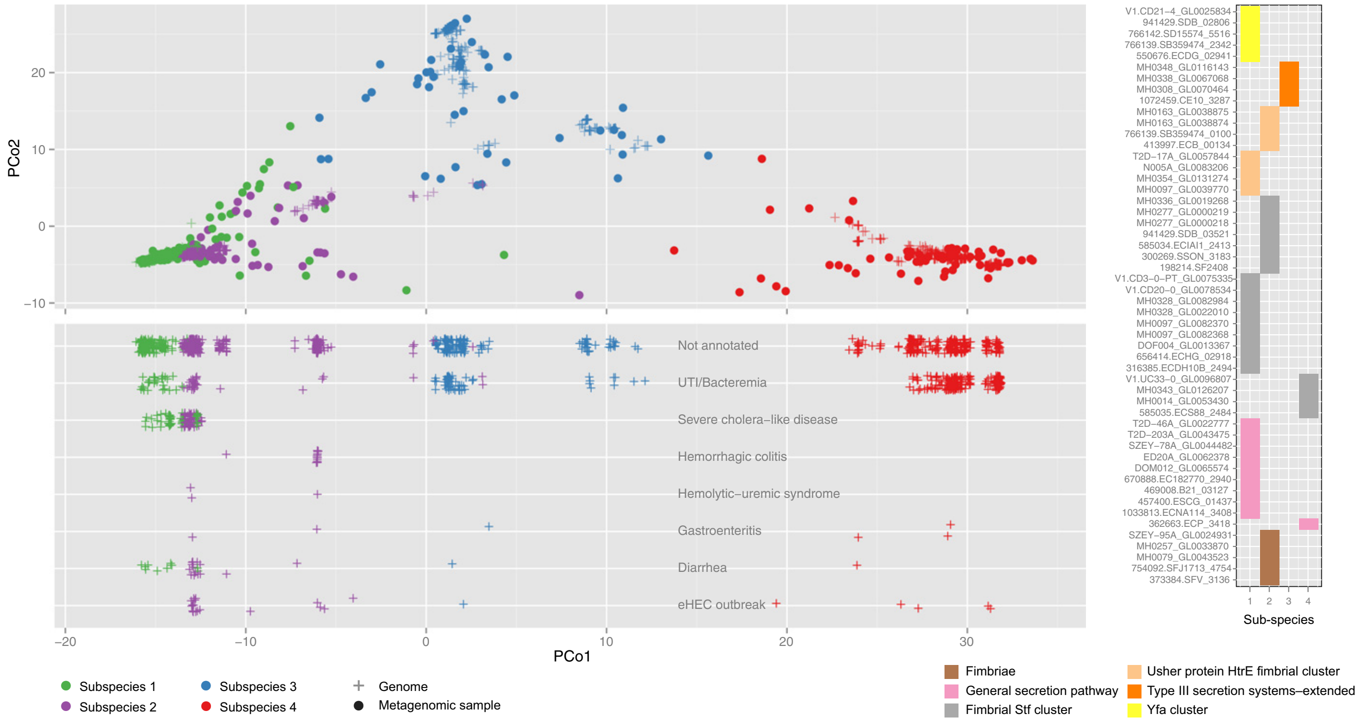

Figure EV4. *Escherichia coli* subspecies.

The four subspecies identified for *Escherichia coli* are visualized in a PCoA plot, in which we additionally placed more than 1,000 reference genomes (crosses; see Materials and Methods). In the bottom panel, the PATRIC annotations for the projected genomes are plotted relative to the first principal coordinate (PCo1), showing a clear enrichment of disease annotations in MGSS1 and MGSS2. Moreover, the eHEC outbreak strains (last row) are strongly enriched in MGSS2, suggesting that commensal and highly pathogenic *E. coli* strains can be very similar in terms of genomic variants. Functional annotations of the SSSCs (right panel) specific to the presumably pathogenic subspecies MGSS1 and MGSS2 show an enrichment of adhesion components in the two subspecies more likely to cause disease.

© 2017 European Molecular Biology Laboratory
